# Numerical Analysis of Dynamic Effects of a Nonlinear Vibro-Impact Process for Enhancing the Reliability of Contact-Type MEMS Devices

**DOI:** 10.3390/s91210201

**Published:** 2009-12-16

**Authors:** Vytautas Ostasevicius, Rimvydas Gaidys, Rolanas Dauksevicius

**Affiliations:** 1 Institute for Hi-Tech Development, Kaunas University of Technology, Studentu 65, LT-51369 Kaunas, Lithuania; E-Mail: vytautas.ostasevicius@ktu.lt; 2 Faculty of Informatics, Kaunas University of Technology, Studentu 50, LT-51368 Kaunas, Lithuania; E-Mail: rimvydas.gaidys@ktu.lt

**Keywords:** MEMS, vibro-impact, contact, finite element analysis, nodal points, vibration modes

## Abstract

This paper reports on numerical modeling and simulation of a generalized contact-type MEMS device having large potential in various micro-sensor/actuator applications, which are currently limited because of detrimental effects of the contact bounce phenomenon that is still not fully explained and requires comprehensive treatment. The proposed 2-D finite element model encompasses cantilever microstructures operating in a vacuum and impacting on a viscoelastic support. The presented numerical analysis focuses on the first three flexural vibration modes and their influence on dynamic characteristics. Simulation results demonstrate the possibility to use higher modes and their particular points for enhancing MEMS performance and reliability through reduction of vibro-impact process duration.

## 1. Introduction

Many traditional devices of microelectromechanical systems (MEMS) do not include contacting surfaces. However in recent years there is an increasing interest in various microsensors and microactuators that employ contact interaction in their normal mode of operation. This trend is determined by the new developments in MEMS technology and new market demands. Among such devices, the fast development of microswitches is very promising. However, insufficient mechanical reliability is one of the main obstacles for wider successful application of these microdevices [1,2]. Interrelated parasitic vibro-impact effects (bouncing) and stiction (a contraction for ‘static friction’) are one of the major reasons that degrade their reliability [1-7]. Due to the elastic response of contacting microstructure of a microswitch, at each on/off cycle, its tip bounces over the substrate a number of times upon contact, as already been reported by K. Petersen in 1979 [8]. This effect is not unexpected, since these switches are essentially a microscopic copy of mechanical relays, in which contact bounce is a well-known phenomenon. It is harmful since it induces pitting and hardening due to the repeated impacts, causes a severe damage of contact surfaces by mechanical hammering and electrical arching (especially during “hot switching” at high current densities), thus promoting the initiation and subsequent propagation of subsurface cracks, facilitating material transfer during detachment of contacting microstructure. Such progressive degradation of the contact interface can eventually lead to stiction and make the device non-functional. Stiction is usually defined as unintentional permanent attachment of compliant microstructure surfaces occurring during contact when restoring elastic forces are unable to overcome adhesive interfacial forces [9-11]. Bouncing degrades device operational speed by increasing actual switching time defined as the time at which a continuous electric current flow can be achieved. MEMS switches must be capable to operate for billions of cycles during their life-time. Limiting of bouncing is crucial since it would increase the reliability and improve their performance by reducing switching time. Many researchers emphasize that in order to achieve these goals a deeper understanding is required in the field of vibro-impact interactions [2,6,7,12,13]. Consequently, to enhance the mechanical reliability of microswitches (like those developed by MEMS research group at Kaunas University of Technology [14]) and other contact-type microdevices, besides a correct selection of the interfacial materials [15], it is of fundamental importance to model and thoroughly analyze characteristic dynamic effects related to complex vibro-impact phenomena. Different research groups throughout the world employ different simulation strategies and numerical models of varying complexity and dimensionality for investigation of contact-type microdevices. The predominant trend is to concentrate modeling efforts on certain aspects of device operation such as electrostatic actuation (e.g., [16]) or viscous air damping (e.g., squeeze-film damping [17]). The other research trend is to pursue development of comprehensive computational models accounting as precisely as possible for all of the major physical processes and coupled-field interactions taking place in operation of contact-type MEMS devices. In this respect some researchers rely on application of classical beam theories with finite difference schemes to model microswitch dynamics by including electrostatic forces, squeeze-film damping and contact bouncing effects [6,7] simulated either by simple linear spring approach [7] or by additionally incorporating adhesive interaction into contact model [6]. The finite element (FE) method is increasingly employed as the multiphysics capabilities of FE software are improving at a rapid pace. A successful example of the latter strategy is a research work by Guo *et al.* [18], where a complex 3-D FE model is developed within ANSYS, accounting simultaneously for electrostatic actuation, squeeze-film damping, modeled by compressible Reynolds equation, and nonlinear contact including adhesion based on Johnson–Kendall–Roberts (JKR) theory. The authors analyze influence of air damping and actuation voltage on bouncing process and demonstrate how modification of the damping and tailoring of the voltage can be used to mitigate the process. Czaplewski *et al.* also applied the FE method for generation of 3-D model of a microswitch including electrostatic actuation but excluding mechanical contact and squeeze-film damping [19]. This approximation is used because the authors focused their attention on electrostatic-structural interaction with a purpose of designing actuation waveform that would completely eliminate contact bouncing. FE analysis is also used by Lishchynska *et al.* in an attempt to simulate bouncing effect in a microswitch [20]. Air damping is not considered by the authors, which simulate electromechanical behavior and propose effective voltage controller scheme for stabilizing off-stage oscillations. However, the authors emphasize that more research work is still required in the field of bouncing reduction in order to achieve stable dynamic behavior during microswitch closure.

A review of the literature on contact bounce in microswitches suggests that extensive research efforts are still needed in this field and that scientific results on underlying dynamical aspects of this detrimental phenomenon are relatively scarce. Modification of electrostatic control mechanism is a predominant approach used for reduction of bouncing however we believe that there is still enough undisclosed potential in the mechanical domain alone, which could be beneficial in tackling the considered problem. Therefore in this paper a contact-type microdevice is analyzed purely from mechanical point of view, thereby concentrating on intrinsic dynamic properties of elastic structures such as natural vibration modes and their advantageous utilization.

## 2. Finite Element Model of Impacting Cantilever Microstructure

**Figure 1.**
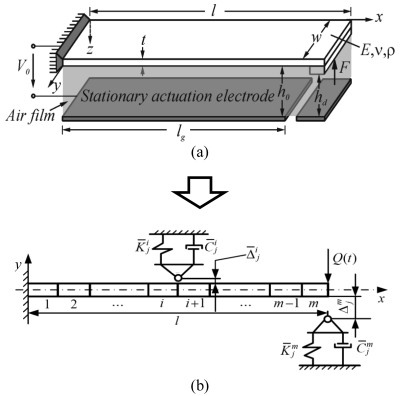
Schematic of: (a) generalized model of common electrostatic contact-type MEMS device operating in ambient air, (b) developed 2-D finite element model of impacting cantilever microstructure.

Figure 1a illustrates a generalized model of common electrostatic contact-type MEMS device operating in ambient air. The device is based on cantilever microstructure, though fixed-fixed configuration is frequent as well. The goal of the current research work is to focus on the impact process alone and carry out detailed investigation of important dynamic aspects of this complex phenomenon. Therefore in this paper electrostatic forces are not considered and it is assumed that the microstructure is operating in vacuum, thus squeeze-film damping is neglected as well (the research of these phenomena have been reported earlier [21-23]). Exclusion of gas environment from the presented numerical model is justified by a preference to avoid ambient gas in device operation since it creates favorable conditions for electrical arching. For simulation purposes a 2-D modeling approach is applied since: a) flexural vibration modes have a much more significant influence on vibro-impact process in comparison to torsional modes and b) it is computationally more cost-effective. Figure 1b presents a schematic of the developed 2-D finite element (FE) model of impacting cantilever microstructure. The following parameter values were used for numerical analysis: microstructure length *l* = 117 μm, width *w* = 30 μm, thickness *t* = 2 μm, Young's modulus, density and Poisson's ratio for Nickel- *E* = 207 GPa, *ρ* = 8,902 kg/m^3^ and *ν* = 0.31 respectively. The model consists of *i =* 1,2,…,*m* linear beam elements located in a single layer and *j =* 1,2,…,*k* motion limiters or supports (0 < *k* < 2 *m*) that are located in *i* = 1,2,…,*m* nodes. Each beam element has two nodes with three degrees of freedom (DOF) at each one (displacement in *x*- and *y*-axis directions and rotation in *x0y* plane). The model was meshed manually with number of finite elements *m* equal to 50, thereby resulting in 150 total DOFs. The sufficiency of this particular mesh density was confirmed by comparative simulations presented in Section 2 and summarized in Figures 4–5. Impact modeling is based on contact element approach and makes use of Kelvin-Voigt (viscoelastic) rheological model, in which linear spring is connected in parallel with a damper–the former represents the impact force and the latter accounts for energy dissipation during impact.

After proper selection of generalized displacements in the inertial system of coordinates, model dynamics is described by the following equation of motion given in a general matrix form:

(1) $$[M]\{\ddot{y}(t)\}+[C]\{\dot{y}(t)\}+[K]\{y(t)\}=\{\begin{array}{l} \{Q(t)\},\text{if}\{{\bar{\Delta }}_{j}^{i}\}>\{y_{i}(t)\}\cap \{\Delta_{j}^{i}\}<\{y_{i}(t)\}\cup f_{i}(y_{i},{\dot{y}}_{i},t)\geq 0;\\ \{Q(t)\}+\{F(y,\dot{y},t)\},\text{if}\{{\bar{\Delta }}_{j}^{i}\}\leq \{y_{i}(t)\}\cup \{\Delta_{j}^{i}\}\geq \{y_{i}(t)\}\cap f_{i}(y_{i},{\dot{y}}_{i},t)<0 \end{array}$$

where [*M*], [*C*], [*K*] are mass, damping and stiffness matrices respectively,{*y*(*t*)}, {*ẏ*(*t*)}, {*ÿ*(*t*)}—displacement, velocity and acceleration vectors respectively. {*Q*(*t*)} is a vector representing the sum of external forces acting on the microstructure. Since external electrostatic and air pressure forces are not considered here, this vector is used as a mechanical load during simulations of free impact vibrations presented in Section 2.

The initial conditions are defined as:

(2) $$\{\dot{y}(0)\}={\dot{y}}^{0},\;\{y(0)\}=\Delta_{j}^{i}$$

where {*F*(*y, ẏ, t*)}—vector of impact interaction between cantilever microstructure and the support. Components *f_i_*(*y, ẏ, t*) represent the reaction of the impacting microstructure and are expressed as:

(3) $$f_{i}(y_{i},{\dot{y}}_{i},t)={\bar{K}}_{j}^{i}\left(\;\left|\Delta_{j}^{i}\right|-\left|y_{i}(t)\right|\;\right)+{\bar{C}}_{j}^{i}{\dot{y}}_{i}(t)$$

where ${\bar{K}}_{j}^{i},{\bar{C}}_{j}^{i}$—stiffness and viscous friction coefficients of the support, $\Delta_{j}^{i}$ —distance from the i-th nodal point of the microstructure to the j-th surface of the support located at the corresponding nodal point. In the case of the considered model the assumption of proportional damping is adequate therefore internal damping is modeled by means of Rayleigh damping approach [24]:

(4) $$\left[C\right]=\alpha_{dM}\left[M\right]+\beta_{dK}\left[K\right]$$

where *α_dM_, β_dK_* are mass and stiffness damping parameters respectively that are determined from the following equations using two damping ratios *ξ*_1_ and *ξ*_2_ that correspond to two unequal natural frequencies of vibration *ω*_1_ and *ω*_2_ [24]:

(5) $$\begin{array}{l} \alpha +\beta \omega_{1}^{2}=2\omega_{1}\xi_{1},\\ \alpha +\beta \omega_{2}^{2}=2\omega_{2}\xi_{2}. \end{array}$$

The presented FE model of the vibro-impact microsystem was implemented in FORTRAN.

## 3. Numerical Analysis of Impact Vibrations of Cantilever Microstructure

Free impact vibrations of elastic microstructures constitute one of the operation modes of contact-type MEMS devices. Complete vibro-impact process consists of free vibrations of the microstructure in the intervals between the impacts and its vibration during the impacts. Therefore, thorough analysis of free and impact vibrations of elastic microstructures is essential. For this purpose special FORTRAN numerical codes were written and used for running detailed dynamic simulations with the developed FE model of the cantilever microstructure that undergoes impacts against the support.

**Figure 2.**
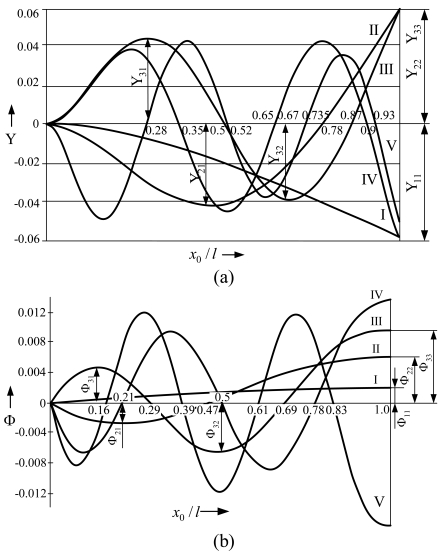
Natural vibration modes of the cantilever microstructure: (a) flexural, (b) rotational. *x*_0_*/l* denotes ratio between the distance *x*_0_ from the anchor of the cantilever and its whole length *l*, Y*_ij_* and Φ*_ij_*—maximum amplitudes of the flexural and rotational modes respectively: index *i*—number of vibration mode, *j*—sequence number of the maximum amplitude point with respect to the anchor point.

The modes of natural transverse vibrations of the microstructure (Figure 2) consist of transverse displacements Y (Figure 2a) and torsions Φ around the axes perpendicular to the plane of vibrations (Figure 2b). The first five modes (I, II, III, IV, V) were obtained, which form nodal points in the intersection with the axis line. These points are denoted by numbers that express the ratio (*x*_0_*/l*) between the distance *x*_0_ from the anchor of the cantilever microstructure and its whole length *l*. The letters Y*_ij_* and Φ*_ij_* denote the values of the maximum amplitudes (deflections) of the flexural and rotational modes.

**Figure 3.**
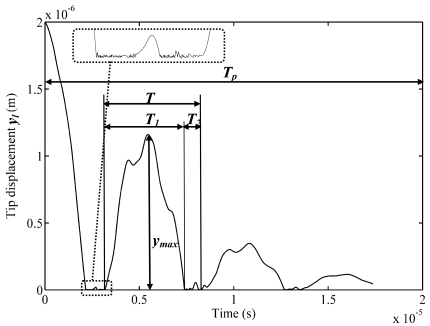
Simulated typical process of free impact vibrations of the cantilever with characteristic parameters: *T_p_*—duration of transient vibro-impact process, *T*—period of free impact vibrations, *T*_1_—duration of vibrations between two impacts, *T*_2_—impact duration, *y_max_*—rebound amplitude.

The process of free impact vibrations of the microstructure for the case when the support is located at the free end of the cantilever is presented in Figure 3. Free impact vibrations were obtained by: (a) displacing free end of the microstructure upwards to a certain height (static analysis) and (b) releasing the microstructure from its statically-deformed position thereby allowing it to impact the support (transient analysis). The obtained complex vibro-impact motion is a result of self-excitation of several vibration modes of the microstructure.

The simulated *y_l_* trajectory was matched to the experimental one by variation of the contact stiffness and damping values in the viscoelastic impact model. Initial guesses of these values were performed empirically based on the available data on material properties of the microstructure and contact surfaces. Thereby the developed FE model of the impacting cantilever was adjusted until an acceptable level of accuracy was achieved. The accuracy of the model was checked quantitatively by using simulated and experimental values of period of free impact vibrations *T* and calculating relative error *δ* = ((|*T*_exp_ − *T*|)/*T*_exp_) × 100. Simulated vibro-impact process in Figure 3 yields *T* ≈ 5.1 μs, while the corresponding measured value is equal to *T_exp_* ≈ 4.9 μs. This gives *δ* ≈ 4%. This discrepancy is sufficiently small and allows us to consider the developed model to be adequate to the physical one.

**Figure 4.**
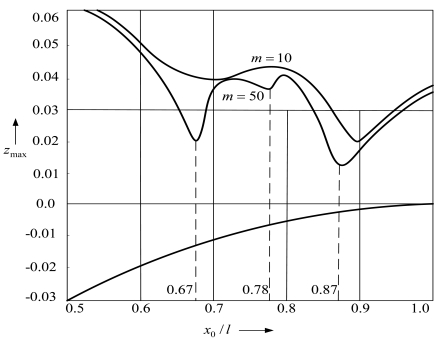
Dependence of dimensionless rebound amplitude of the microstructure *z*_max_ = *y*_max_/*l* on the position of the support expressed as a ratio between the distance *x_0_* from the anchor of the cantilever and its whole length *l*.

Temporal characteristics that are most typical for the free impact vibrations are: *T_p_*—duration of the transient vibro-impact process, *T*—period of free impact vibrations, *T*_1_—duration of vibrations between two impacts, *T*_2_—impact duration. The accuracy of simulation results is significantly influenced by the density of the finite elements mesh. Figure 4 presents the dependence of the maximum amplitude of the post-impact rebound *z*_max_ = *y*_max_/*l* on the position of the support for the FE mesh having different number of finite elements *m*. When *m* = 50, the obtained minimums of rebound amplitudes obviously coincide with the nodal points of some vibration modes, which is not so obvious for *m* = 10.

Figure 4 reveals that the smallest rebound amplitudes are obtained when the support is located in points coinciding with *x*_0_/*l* = 0.87 or *x*_0_/*l* = 0.67. A slight decrease in the rebound amplitude is also observed at *x*_0_/*l* = 0.78. The lower curve in Figure 4 that asymptotically approaches the axis line corresponds to the deflection of the free end of the microstructure during the impact with the support.

**Figure 5.**
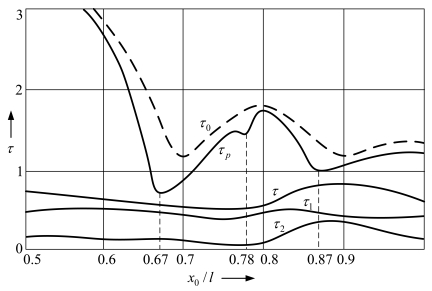
Temporal characteristics of free impact vibrations of the microstructure as a function of support position: *τ_p_* = *ω*_1_*T_p_* (for *m* = 50), *τ_0_* = *ω*_1_*T_p_* (for *m* = 10), *τ* = *ω*_1_*T, τ*_1_ = *ω*_1_*T*_1_, *τ*_2_ = *ω*_1_*T*_2_. *ω*_1_—first circular natural frequency of the cantilever.

Figure 5 illustrates temporal characteristics of free impact vibrations in the case of different FE mesh density. It indicates that when the support is placed in points *x*_0_/*l* = 0.87, 0.78, 0.67, the duration of transient vibro-impact process *τ_p_* = *ω*_1_*T_p_* (*ω*_1_ - first circular natural frequency of the cantilever) may be reduced. The remaining characteristics are less sensitive to variations of support position.

The points *x*_0_/*l* = 0.78, 0.87 coincide with the nodal points of the 2^nd^ and the 3^rd^ flexural vibration modes, while *x*_0_/*l* = 0.67—with the maximum amplitude point of the 3^rd^ mode. These points will be referred to as particular points of natural vibration modes. The subsequent numerical analysis will be confined to the consideration of the first three modes since they significantly influence the dynamic characteristics of the vibro-impact process. In order to clarify the nature of these characteristics it was necessary to determine vibration modes of the microstructure during the impact on the support.

**Figure 6.**
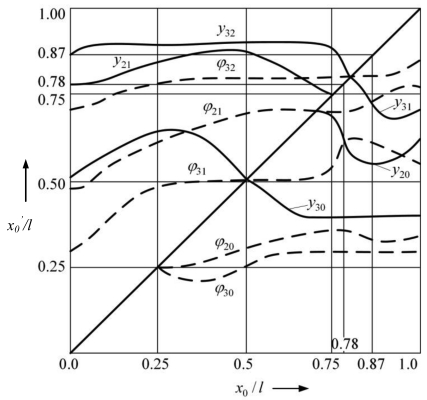
Dependence of nodal points of the displacement (*y_ij_*) and rotational (*ϕ_ij_*) vibration modes of the supported cantilever microstructure on the position of the support: *i*—number of vibration mode, *j*—sequence number of the nodal point with respect to the anchor point of the microstructure.

Figure 6 provides dependencies of the location of nodal points of the modes on the position of the support for the case of supported microstructure. The nodal points *y_ij_* and *ϕ_ij_* of the displacement mode Y*_i_* and rotational vibration mode Φ*_i_* are designated by two indices: *i* refers to the number of vibration mode, *j*—to the sequence number of the nodal point with respect to the anchor point of the microstructure. In comparison to unsupported microstructure, an additional nodal point (*i* = 0) is added to each mode for the case of supported microstructure. In Figure 6 the diagonal line represents the shifting of the support from the anchor of the microstructure to the free end. It is obvious that the position of the support determines the position of nodal points of the mode. When the support is located at the anchor, the modes and nodal points coincide with those of the unsupported microstructure, which is demonstrated by the nodal points indicated on the vertical axis *x_0_*′*/l*. This distribution of locations of nodal points characterizes the microstructure before the impact. However, when the support is shifted, portion of the nodal points of the displacement modes Y*_i_* are shifted together, though this is not characteristic for rotational modes. This phenomenon is related to the pin-joint support of the microstructure.

Simulated curves presented in Figure 6 enable explanation of the cause of changes in the dynamic characteristics of the considered vibro-impact microsystem in that case when the support is located in point *x*_0_/*l* = 0.87: the 2^nd^ nodal point of the 3^rd^ vibration mode of the supported microstructure coincides with the same point for the case of unsupported one (*x*_0_/*l* = 0.87). This implies that in the process of impact vibrations this point does not change its position either before or during the impact. This mode is amplified when the force of impact is applied to this nodal point. Consequently, the amplitude of the 3^rd^ mode increases resulting in more intensive energy dissipation in the material of the microstructure since it is considered [24] that energy dissipated by the structure that vibrates in the higher mode exceeds energy dissipated by the structure vibrating in its fundamental mode as many times as is the ratio of natural frequencies of the modes. Thus, the energy dissipated in the microstructure that vibrates in the fundamental mode is nearly *ω*_3_/*ω*_1_ ≅ 17 times less than in the case of vibrations in the 3^rd^ mode. It is evident that intensification of the amplitude of the 3^rd^ mode by locating the support at its nodal point does not cancel the first two modes. The fact that nodal points *y*_31_ and *Φ*_21_ coincide in the case when the support is located at point *x*_0_/*l* = 0.87 suggests the possibility of amplification of the 2^nd^ mode as well. However, the advantages achieved in the considered case are first of all related to the intensification of the amplitude of the 3^rd^ vibration mode (during vibro-impact process cantilever vibrations in a wide frequency range are excited). The advantages achieved when the support is positioned in point *x*_0_/*l* = 0.78 are related to the intensification of the 2^nd^ vibration mode amplitude because this is the point in which the nodal points of the 2^nd^ vibration mode of the supported and unsupported microstructure are located (*x*_0_/*l* = 0.78). As Figure 6 indicates, the intersection of the trajectories of nodal points *y*_20_ and *Φ*_31_ when the support is located in point *x*_0_/*l* = 0.78 also enables to intensify amplitude of other flexural modes.

**Figure 7.**
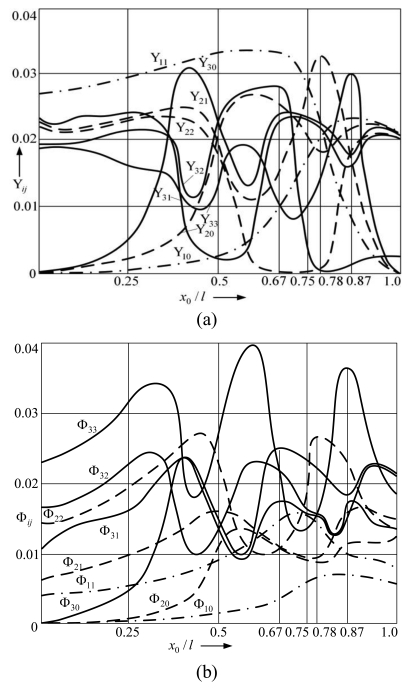
Dependence of maximum amplitudes of the flexural (Y*_ij_*) and rotational (Φ*_ij_*) modes on the position of the support: (a) flexural, (b) rotational. Index *i*—number of vibration mode, *j*—sequence number of the maximum amplitude point with respect to the anchor point.

The presented explanation is also confirmed by the dependences of the maximal amplitude points of separate vibration modes on the position of the support (Figure 7). The relationship of amplitudes of displacement modes Y_11_ with respect to support locations (Figure 7a) demonstrates that when the support is located in point *x*_0_/*l* = 0.87, the amplitude of the 3^rd^ displacement mode Y_33_ is maximal whereas other amplitudes do not reach their maximal values in this point. Positioning of the support in the point of the maximum amplitude of the 3^rd^ vibration mode (*x*_0_/*l* = 0.67) amplifies the displacement amplitude Y_32_ that coincides with the said point of maximum amplitude.

Amplitudes Y_30_ and Y_31_ are increased as well, whereas amplitude Y_33_ is reduced. When the support is positioned in the nodal point of the 2^nd^ displacement mode, the displacement amplitude Y_22_ increases whereas other amplitudes of the 2nd mode decrease. Similarly, the amplitudes of rotational vibration modes Φ_11_ are intensified as well (Figure 7b). Due to the impact of the cantilever on the support located in one of the particular points of vibration modes, the associated amplitudes increase even further thereby amplifying separate vibration modes.

**Figure 8.**
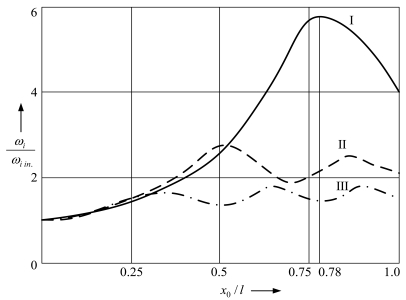
Dependences of the ratio between the circular natural frequencies of the supported microstructure *ω_i_* and those of the unsupported one *ω_iin_* on the position of the support (*i* = I, II, III).

After the performed analysis of the behavior of the nodal points and the points of maximum amplitude with respect to the support location, it is important to investigate the dependence of the frequencies of separate vibration modes on the position of the support. Figure 8 illustrates simulated dependences of the ratio between circular natural frequencies of the supported microstructure *ω_i_* and those of the unsupported one *ω_iin_*. It may be observed that the 1^st^ natural frequency of the supported microstructure reaches the maximum value when the support is located in point *x*_0_/*l* = 0.78 whereas the 2^nd^ and the 3^rd^ natural frequencies reach their maximum values when the support is located in other positions.

Therefore, in order to ensure maximum vibrational stability of a contact-type MEMS device containing a supported cantilever microstructure, the support must be positioned in point *x*_0_/*l* = 0.78. In this case the resonance frequency of the microsystem is maximum and, additionally, it becomes possible to amplify the amplitudes of the 2^nd^ mode of natural vibrations and to dissipate a significant portion of kinematically-transferred energy in the material of the microstructure. Furthermore, when the support is located in point *x*_0_/*l* = 0.78, the difference between the 1^st^ and the 2^nd^ natural frequencies of the supported microstructure is maximum, and by selecting the stiffness of the support to be located in the given point, the 1^st^ natural frequency may be brought closer to its 2^nd^ natural frequency thereby increasing its vibrational stability under external kinematical excitation, which may be very important when microdevice is located on the moving object.

**Figure 9.**
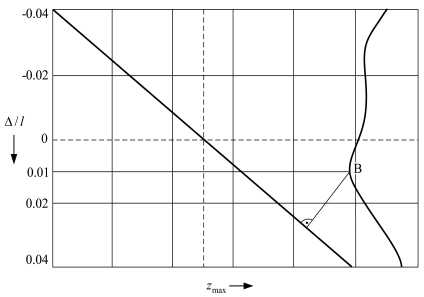
Dependence of maximum rebound amplitudes of the cantilever microstructure *z*_max_ = *y*_max_/*l* on the magnitude of dimensionless prestress Δ/*l*. Δ refers to prestress, *i.e.*, distance of the displacement of the cantilever free end in the direction perpendicular to the contact surfaces.

Common contact-type MEMS devices incorporate gaps between compliant and fixed microstructures. However, feasible MEMS designs may be also based on usage of prestress of contacting links. Therefore it is crucial to select the prestress in such a way that minimal rebound amplitudes are achieved resulting in reduced energy consumption during device control. Figure 9 presents simulated maximum rebound amplitudes *z*_max_ = *y*_max_/*l* as a function of prestress Δ/*l*, when the support is located at the free end of the cantilever microstructure. The diagonal line indicates the position of the support when it is vertically moved from the boundary position to the position of maximum prestress. The dashed lines at zero level represent the equilibrium position of the microstructure (vertical) and zero prestress (horizontal).

As the simulation results in Figure 9 demonstrate, minimum rebound amplitudes with respect to the equilibrium position are characteristic in the case of small prestress magnitudes (point *B*, when Δ/*l* = 0.01). By drawing a perpendicular from point *B* to the diagonal line, minimum amplitudes of the microstructure rebound are determined. Thus, in order to obtain the smallest bouncing that ensures minimum power consumption, the prestress should be selected in accordance to point *B*.

**Figure 10.**
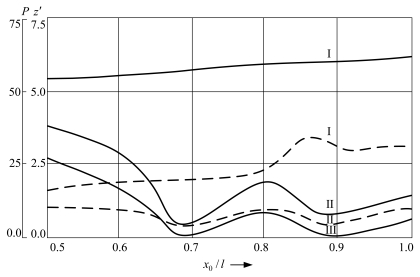
Dependence of impact velocity *z*′ (continuous lines) and contact pressure force *P* (dashed lines) on the position of the support.

In addition to the amplitude-frequency characteristics of free impact vibrations, it is essential to determine the velocities and the forces induced during the impact. Figure 10 demonstrates the dependence of the pre-impact velocity (continuous lines) and original contact pressure force *P* (dashed line) on the position of the rigid support at zero prestress during the first three impacts (I, II, III) of the microstructure on the support. When the support is located in the particular points of the 3^rd^ flexural mode of the cantilever microstructure, a decrease in the velocity and original contact pressure force is observed, which is related to the increase in the dissipated energy in the material.

**Figure 11.**
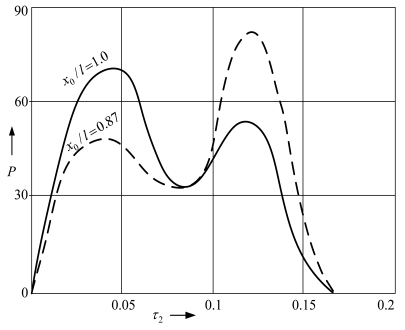
Dependence of contact pressure force *P* of the cantilever microstructure on the position of the support. *τ*_2_ = *ω*_1_*T*_2_, *T*_2_—impact duration, *ω*_1_—first circular natural frequency of the cantilever.

Simulations results (Figure 11) also reveal that during the microstructure impact on the support positioned in point *x*_0_/*l* = 0.87 the contact pressure force is lower in the first stage of impact than in the second one, as compared with the opposite characteristics of the pressure force when the support is located at *x*_0_/*l* = 1.

## 4. Conclusions

In this paper we have presented a 2-D finite element model of cantilever microstructure impacting against viscoelastic support thereby representing a general case of contact-type MEMS devices. The model was developed within FORTRAN environment. Impact is modeled by means of contact-element approach that uses Kelvin-Voigt rheological element taking into account both contact stiffness and damping. Values of these parameters were selected empirically to match experimentally-obtained vibro-impact trajectories. Results of numerical analysis of characteristic vibro-impact process–free impact vibrations–were reported by considering three stages of the studied process: pre-impact, impact and post-impact. Obtained numerical results are provided in a dimensionless form and therefore are applicable across all scales ranging from macro to nano.

Numerical analysis is centered around the consideration of the first three flexural modes of the cantilever microstructure since they have a major effect on dynamic characteristics of the vibro-impact process. Investigation of influence of support position (along horizontal axis of the microstructure) on maximum post-impact rebound amplitudes indicates that the smallest values are obtained when the support is located in specific points coinciding with the nodal points of the 2^nd^ and the 3^rd^ flexural vibration modes (*x*_0_/*l* = 0.78 and 0.87 respectively) as well as with the amplitude peak of the 3^rd^ mode (*x*_0_/*l* = 0.67). Simulations reveal that support (contact point) positioning in these so-called particular points of vibration modes results in reduction of transient vibro-impact process thereby enabling to increase MEMS device operational speed as well as to enhance its reliability by diminishing detrimental consequences of this process. In-depth numerical analysis was conduced in order to reveal the physical nature of the aforementioned findings. For this purpose vibration modes of the microstructure during the impact on the support were determined. It is known that the position of the support determines the position of nodal points of the mode: when the support is shifted, portion of the nodal points of the flexural modes are shifted together. However it was revealed that in the process of impact vibrations the aforementioned particular points do not change their position either before or during the impact. This implies that the 2^nd^ and 3^rd^ modes are amplified when the force of impact is applied to these points. The effect is particularly pronounced in the case of the 2^nd^ nodal point of the 3^rd^ flexural mode (*x*_0_/*l* = 0.87). Consequently, the amplitude of the 3^rd^ mode increases resulting in more intensive energy dissipation in the material of the microstructure (energy dissipated is *ω*_3_ /*ω*_1_ ≅ 17 times larger than in the case of microstructure vibrating in its fundamental mode). Increase of dissipated energy in the material at this particular point is also confirmed by observed reduction of the induced velocity and contact pressure force during impact.

Numerical study of influence of support position on the natural frequencies of separate vibration modes indicates that maximization of vibrational stability of contact-type MEMS devise containing supported microstructure is achieved by placing support at *x*_0_/*l* = 0.78 due to maximization of the 1^st^ natural frequency of the supported microstructure. By selecting the stiffness of the support to be located in the given point, the 1^st^ natural frequency may be brought closer to its 2^nd^ natural frequency thereby increasing the vibrational stability.

Obtained results of numerical analysis reveal huge potential of advantageous usage of higher vibration modes with their particular points for suppressing harmful bouncing process in contact-type microdevices resulting in improved reliability and performance. Therefore further research efforts are necessary in this field in order to identify different approaches for control of impact-related processes thereby enabling designers to develop innovative MEMS sensors and actuators that operate in contact mode.
